# Genome-Wide Association Study of Yield-Related Traits and Photoperiod Response in Rice

**DOI:** 10.3390/plants15060875

**Published:** 2026-03-12

**Authors:** Ziming Zang, Chang Liu, Zhaoqin Wang, Cheng Fan, Juncong Chen

**Affiliations:** 1 School of Finance, Nanjing Agricultural University, Nanjing 210095, China; 2 College of Sciences, Nanjing Agricultural University, Nanjing 210095, China

**Keywords:** QTN, yield traits, photoperiod, candidate genes, small-effect loci

## Abstract

Yield-related traits of rice (*Oryza sativa* L.) are pivotal to safeguarding global food security. As a powerful and efficient strategy, genome-wide association study (GWAS) has identified numerous genes for yield-related traits in rice over recent decades, providing critical resources for germplasm improvement. Most yield-related traits are complex quantitative traits controlled by multiple genes with diverse effect sizes, and traditional GWAS approaches have limited power to detect small-effect loci. In this study, we employed Fast3VmrMLM, a compressed mixed linear model integrating genome-wide scanning and machine learning, to perform GWAS for 10 key yield-related traits using a panel of 529 rice accessions and 4,945,006 single-nucleotide polymorphisms (SNPs). The traits included heading date, plant height, panicle number, effective panicle number, yield per plant, spikelet length, grain length, grain width, grain weight, and grain thickness. We detected 141 significant quantitative trait nucleotides (QTNs) associated with target traits and identified 92 previously validated genes located near these QTNs. As a key environmental regulator, photoperiod directly controls flowering and indirectly modulates yield-related traits, and we further identified 182 photoperiod-responsive candidate genes via differential expression and Gene Ontology (GO) enrichment analysis. Through tissue-specific expression analysis, homology analysis with *Arabidopsis* genes, and haplotype-phenotype differential analysis, six pleiotropic candidate genes were confirmed; notably, *LOC_Os02g02210* appears to contribute substantially to grain width and yield-related traits. In conclusion, Fast3VmrMLM proved effective for dissecting the genetic basis of yield-related traits, especially in detecting small-effect loci. These results not only establish a potential genetic link between photoperiod regulation and rice yield formation but also provide high-confidence candidate genes and loci that will accelerate functional genomic studies and precision molecular breeding for high-yield rice.

## 1. Introduction

Rice (*Oryza sativa* L.) is a globally vital food crop that serves as the staple food for more than half of the world’s population. It plays an irreplaceable core role in ensuring food security, supporting social stability, improving nutritional status, and promoting development [1,2]. Over the past decades, driven by the continuous effects of rice cultivars improvement, the global total rice yield has achieved remarkable growth, increasing from 547 million tons in 1994 to 787 million tons in 2023 (http://www.fao.org/faostat/en/#data/QC/visualize, accessed on 15 November 2025), and this growth trend is particularly pronounced in major rice-producing countries. However, rice yield is confronted with dual challenges of resource constraints and diminishing marginal returns, rendering a bottleneck for further yield increase. In the future, with the continuous growth of the global population, the rigid demand for rice is projected to expand further [3], which imposes an urgent requirement for the sustained enhancement of rice production capacity.

Beyond its role as a primary staple food, rice also serves as a pivotal model crop for plant science research and genetic improvement. Its inherent characteristics, including a relatively small genome, ease of genetic transformation, and plant regeneration capabilities, make it an ideal experimental system for understanding the genetic mechanisms of complex quantitative traits and conducting molecular breeding research [4]. Although extensive genomic resources are available for rice, including multiple high-quality reference genomes, large-scale germplasm banks, and various public databases, elucidating the genetic basis of complex traits remains challenging. Traditional quantitative trait locus (QTL) mapping has provided valuable insights into the genetic architecture of these traits but is limited by mapping population design and resolution. In contrast, a genome-wide association study (GWAS) offers a complementary strategy with higher mapping resolution, enabling a more precise dissection of complex quantitative traits. Continuously deepening the genetic dissection of key traits in rice holds profound strategic significance for overcoming breeding bottlenecks.

GWASs are extensively employed for the screening of genetic variations underlying complex traits in large-scale germplasm populations [5]. In the fields of yield-related agronomic traits, including heading date, plant height, and 1000-grain weight, numerous studies have adopted single-locus GWAS methodologies for the detection of genetic variations, including the mixed linear model (MLM) [6,7], efficient mixed-model association expedited (EMMA) [8], and Genome-wide Efficient Mixed-Model Association (GEMMA) approach [9]. In recent years, a multitude of genes associated with quantitative traits have been successfully identified and validated in rice through GWAS. For instance, *OsSPL13*, a gene tightly associated with seed shape, has been verified as regulating the length and yield [10]. Genes *OsGS3* and *OsGW2* were identified via GWAS as major-effect genes controlling seed size and width, respectively [11,12]. Furthermore, the *sd1* gene, selected as a semi-dwarf trait from varieties like DGWG, encodes gibberellin 20 oxidase and is associated with reduced plant height and enhanced lodging resistance. As a key driver of the rice Green Revolution [13], it produces lodging-resistant, high-yield varieties, enabling higher nitrogen input and a 20–30% yield increase. Later, *sd1* was redetected and independently validated by GWAS [14,15], which demonstrates its strength in mining important loci. Such GWAS-driven validations not only reinforce the genetic basis of important traits but also provide precise targets for molecular marker-assisted selection and genetic improvement.

Among the diverse environmental factors affecting complex traits in rice, photoperiod emerges as a crucial regulator that directly controls flowering date and indirectly impacts yield traits. Previous studies have predominantly focused on the photoperiod response of major-effect genes during the heading stage. Nevertheless, a systematic and in-depth dissection of the genetic mechanisms underlying photoperiod-mediated regulation of other yield-related traits via minor-effect gene networks remains lacking, particularly the pleiotropic roles of these minor-effect genes in natural variation populations [16]. In the development of GWAS methodologies, existing approaches typically rely on genome-wide scanning to identify loci with strong signals. While these methods incorporate additive effects and polygenic backgrounds within a mixed-model framework, and can be extended to include dominant effects, their primary focus remains on detecting major-effect loci. This limitation results in inadequate statistical power for detecting small-effect variants and rare variants [17,18,19]. To overcome the limitations of traditional single-gene GWAS methods in detecting minor small-effect QTNs and dissecting polygenic backgrounds, as well as to elucidate the regulatory mechanisms of photoperiod on complex traits, we utilized a compressed mixed model with three variance components, Fast3VmrMLM [20]. This model employs a two-stage framework integrating genome-wide scanning and machine learning: the first stage screens potential associated markers using a relatively lenient threshold, whereas the second stage identifies significantly associated loci in a multi-locus model via an empirical Bayesian approach.

In this study, we conducted yield-related traits’ genetic dissection using 529 rice germplasm resources with extensive genetic diversity and their high-density single-nucleotide polymorphisms (SNPs). Notably, this study focused on photoperiod as a core environmental variable. By integrating differential expression analysis with functional enrichment analysis, we aimed to detect candidate genes responsive to photoperiod stress and mine key genetic loci with pleiotropic effects on multiple yield traits.

## 2. Materials and Methods

### 2.1. The 529 Rice Germplasm Panel

The rice germplasm panel used in this study consists of 529 germplasm resources of Asian cultivated rice. This germplasm panel encompasses rice materials from 73 countries worldwide, including major subspecies types such as Xian/indica (indica rice), Geng/japonica (japonica rice), and Aus (winter rice), as well as a substantial number of intermediate materials, systematically representing the genetic diversity of cultivated rice in Asia (Table S3). All samples underwent whole-genome resequencing using the Illumina HiSeq 2000 platform with the 90 bp paired-end sequencing strategy (>2.5× per genome), generating approximately 6.7 billion high-quality reads. Following bioinformatics analysis procedures, including sequence alignment, variant detection, and genotype imputation, high-density genotype data were ultimately obtained for each sample [21]. The panel was constructed with consideration for both genetic diversity and breeding application value.

### 2.2. Phenotypic Data and Statistical Analysis

We re-analyzed 10 key yield-related traits across a panel of 529 rice (*Oryza sativa* L.) germplasm resources and 4,945,006 SNPs [21,22] using the Fast3VmrMLM [20] to identify significant SNPs, that is, QTNs for 10 yield-related traits. Phenotypic datasets were downloaded from the Rice Diversity Database (https://ricevarmap.ncpgr.cn, accessed on 17 October 2025). The yield-related traits included heading date (HD), plant height (PH), number of panicles (NP), number of effective panicles (NEP), yield (Y), spikelet length (SL), grain length (GL), grain width (GW1), grain weight (GW2), and grain thickness (GT) [23,24]. To visualize all 10 traits, descriptive statistical analyses (mean, minimum, maximum, range, standard deviation, and coefficient of variation) and Pearson correlation analyses were performed on each phenotypic dataset by using R version 4.1.2 (https://www.r-project.org/ accessed on 19 November 2025).

### 2.3. Genotypic Data

**Figure 1:**
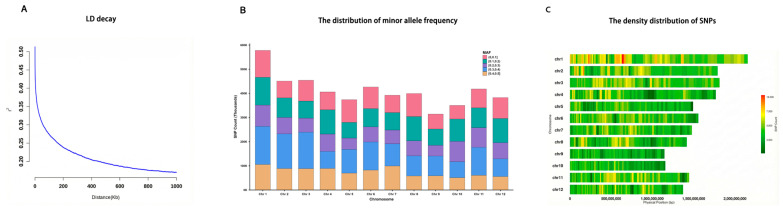
The genetic information of SNPs and the distribution of QTNs and genes across all chromosomes. (**A**) Linkage disequilibrium decay plot for high-quality SNPs. (**B**) The distribution of MAF. (**C**) The density distribution of SNPs.

Genotype data for 529 rice accessions were obtained from the Rice Diversity Database (https://ricevarmap.ncpgr.cn, accessed on 17 October 2025). This dataset contains 17,397,026 high-quality SNP arrays evenly distributed across 12 rice chromosomes. We subsequently performed quality control on the SNP dataset using PLINK (Version 1.9), filtering out markers with minor allele frequency (MAF) < 0.05 and a genotype missing rate > 15%. The imputation of missing markers was conducted using Beagle (Version 5.4) [21,22]. Ultimately, 4,945,006 high-quality SNPs with known physical positions were obtained for subsequent analyses. To visualize the genotypes in this study, we used PopLDdecay (Version 3.31, https://github.com/BGI-shenzhen/PopLDdecay accessed on 19 November 2025) to calculate linkage disequilibrium for SNPs within a 200 kb window (Figure 1A). Additionally, the CMplot package in the R program was employed to plot the distribution of 4,945,006 SNPs across the 12 chromosomes. Figure 1B,C illustrate the distributions of minor allele frequencies and the locus density on each chromosome, respectively. These loci exhibited an approximate uniform distribution, indicating that the dataset is suitable for rice genetic dissection.

### 2.4. GWAS

GWAS was performed to identify QTNs using the Fast3VmrMLM v1.0 package in R (https://github.com/YuanmingZhang65/Fast3VmrMLM accessed on 19 November 2025) [20], a computationally efficient tool optimized for large-scale genetic analysis. For the marker scanning stage, variants with a Bonferroni-corrected *p*-value ≤ 0.01/*m* (where *m* denotes the total number of markers) were considered potentially associated, and then QTNs were further identified by EM empirical Bayes with a significance threshold of a LOD score ≥ 3.0 [17,20] in the last full model. For comparison, we also performed IIIVmrMLM in R (https://github.com/YuanmingZhang65/IIIVmrMLM accessed on 19 February 2026) [18] to identify QTNs. The thresholds for significant QTNs were the same as Fast3VmrMLM.

### 2.5. SNP Annotation and the Identification of Known Genes

Significant QTNs associated with the target traits were detected using the Fast3VmrMLM method. To identify candidate genes and mine novel potential candidate genes, we annotated genes flanking all identified QTNs based on the linkage disequilibrium decay plot (Figure 1A) [25,26], using the China Rice Data Center database (RICEDATA, https://ricedata.cn/, accessed on 19 November 2025). RICEDATA is a comprehensive, expertly curated repository for rice functional genomics, integrating extensive datasets including gene annotations, mutant phenotypes, and experimentally validated gene functions linked to rice agronomic traits. Based on the linkage disequilibrium decay threshold (within a 200 kb window flanking each QTN), we further systematically screened and identified previously confirmed genes, which were experimentally verified to modulate rice yield formation, growth, and development in prior studies.

### 2.6. Functional Enrichment Analysis and the Identification of Candidate Genes

In addition to the known genes associated with the target traits, differential expression analysis was performed on the GSE163030 dataset [16] from the Gene Expression Omnibus (GEO; https://www.ncbi.nlm.nih.gov/geo/ accessed on 19 November 2025) to identify photoperiod-responsive differentially expressed genes (DEGs) among unreported genes.

The used material was the japonica rice cultivar ‘9522’ (*Oryza sativa* L. ssp. *japonica* cv. 9522), which was cultivated in paddy fields in Shanghai, China, during the growing season from June to August. Photoperiod treatments were initiated at the young panicle differentiation stage and continued until the end of sample collection. Long-day (LD) conditions were achieved with natural light, resulting in a photoperiod of approximately 14 h; short-day (SD) conditions were achieved by covering the plants with a shade cloth 2 h before sunset, resulting in a photoperiod of 12 h. Anther samples were collected according to developmental stages: anthers in mitotic phase I (anther length approximately 2.2 mm, corresponding to stage 11) were sampled at six time points (00:00, 04:00, 08:00, 12:00, 16:00, and 20:00) during the day, with two biological replicates established at each time point [16]. Two types of differential expression analyses were performed: one was the inter-group comparisons between the LD and SD treatments during mitotic phase I [16], and the other was the intra-group comparisons between adjacent time points within the same treatment.

The limma package in R (Version 3.66.0) was utilized to screen for photoperiod-responsive DEGs with the criteria of |log2FoldChange| > 1 and false discovery rate-adjusted *p* < 0.05. Subsequently, these photoperiod-responsive DEGs were cross-verified with the previously detected trait-associated genes, resulting in the identification of multiple abiotic stress-responsive DEGs related to yield-related traits.

For Gene Ontology (GO) functional enrichment analysis, the above-mentioned trait-associated DEGs were analyzed using the online tool agriGO v2.0 (https://systemsbiology.cau.edu.cn/agriGOv2/ accessed on 19 November 2025) [27]. Significantly enriched GO terms were identified via singular-gene enrichment analysis and Fisher’s exact test, with a significance threshold of *p* < 0.05 [28].

### 2.7. Blast of Homologous Genes in Arabidopsis

Homologous genes in *Arabidopsis thaliana* were identified using Ensembl Plants (https://plants.ensembl.org/Oryza_sativa/ accessed on 4 December 2025) and the BLAST (https://plants.ensembl.org/Oryza_sativa/Tools/Blast accessed on 4 December 2025). This homology analysis was used for functional annotation.

### 2.8. Analysis of Haplotype and Phenotypic Difference

To validate the association loci between candidate genes and target traits, the geneHapR package in R (https://github.com/ZhangRenL/geneHapR accessed on 10 December 2025) [29] was employed to perform linkage disequilibrium and haplotype block analyses, as well as to estimate the haplotype frequencies of pleiotropic genes among the candidate gene set. For each gene, significant variants were used for haplotype delineation, and phenotypic differences between haplotypes were assessed via the *t*.test function in R with a significance level of *p* < 0.05.

## 3. Results

### 3.1. Phenotypic Variation

Phenotypic statistics of 10 yield-related traits were performed across 529 rice accessions to detect the significant genetic variation. Descriptive statistics for all traits are presented in Table 1. Wide phenotypic variation was observed for all traits, with distinct variation patterns among different traits.

Yield per plant exhibited the largest phenotypic variation among all traits, with a CV of 0.45 (Table 1). Its phenotypic values ranged from 4.41 g to 92.66 g, with a mean of 31.03 g, indicating a wide range of yield differences among the rice accessions. The second highest variation was observed in the NO. of panicles and the NO. of effective panicles, both with a CV of 0.43. The number of panicles ranged from 3.00 to 47.20, with a mean of 13.32; the number of effective panicles varied from 3.00 to 45.80, with a mean of 13.14, suggesting significant differences in panicle-related traits among the population. By contrast, heading date, grain weight, spikelet length, grain width, and grain length showed moderate to relatively low CV values, indicating more stable phenotypic performance across the population. Grain thickness exhibited the smallest phenotypic variation, with the lowest CV of 0.07. Its phenotypic values were relatively concentrated, ranging from 1.58 to 2.45, with a mean of 2.00, indicating that grain thickness was a relatively stable trait in rice accessions. Overall, the phenotypic diversity observed in this population provides a solid foundation for subsequent genome-wide association analysis.

**Figure 2:**
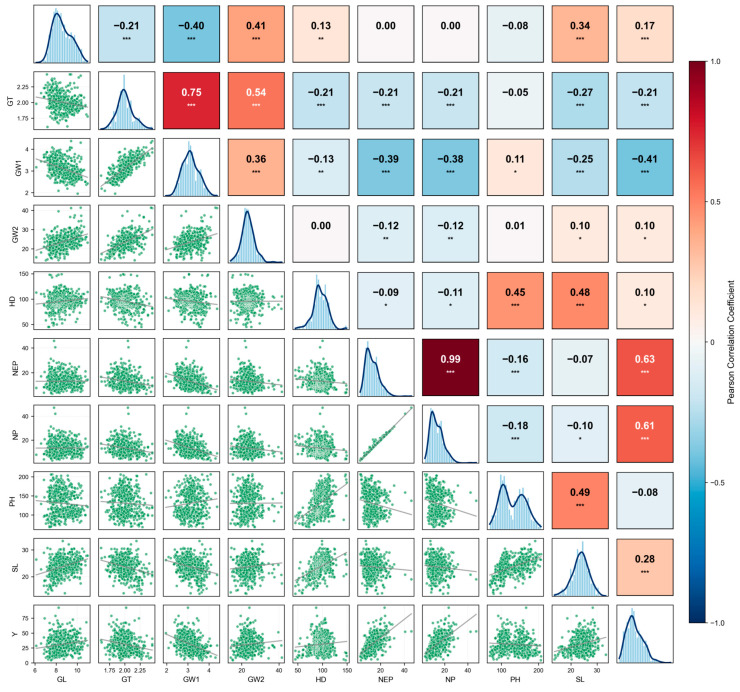
Distribution of 10 rice yield traits and Pearson correlation coefficient. The diagonal histogram represents the distribution of each trait. Linear regression statistics between the two traits are below the diagonal, while the correlation coefficients are above the diagonal. Traits are Grain thickness (GT); Grain width (GW1); Grain weight (GW2); Heading date (HD); Number of effective panicles (NEP); Number of panicles (NP); Plant height (PH); Spikelet length (SL); and Yield (Y). The number of stars represents the result of *t* test at different significance levels (*: *p* < 0.05; **: *p* < 0.01; ***: *p* < 0.001).

To further clarify the phenotypic relationships among these 10 yield-related traits, a pairwise correlation plot was constructed (Figure 2), which is a typical phenotypic correlation analysis tool used to display the linear relationships between multiple quantitative traits. This plot consists of three parts: the diagonal represents the phenotypic distribution histogram and density curve of each trait, showing the variation in the trait in the population. The lower triangle indicates the scatter plots between each pair of traits, intuitively presenting the trend of their linear relationship. The upper triangle indicates the Pearson Correlation Coefficient of each pair of traits, where colors and values together represent the strength and direction of the correlation—red indicates positive correlation (the larger the value, the stronger the positive correlation), while blue indicates negative correlation (the smaller the value, the stronger the negative correlation). 

As presented in Figure 2, a nearly perfect positive correlation was shown between NEP and NP (*r* = 0.99), which is consistent with biological expectations since effective panicles are the core component of total panicles. Meanwhile, Y was significantly positively correlated with both NEP (*r* = 0.63) and NP (*r* = 0.61), indicating that panicle number, especially effective panicle number, is a key positive factor determining single-plant yield. However, most traits had weak correlations with GW2 (*r* close to 0), which may be related to low measurement repeatability or heritability of this trait. HD (heading date) also had weak correlations with NEP/NP (panicle number) and Y (yield) (*r* ≈ 0.1).

Overall, the abundant phenotypic diversity observed in this population provides a solid foundation for subsequent GWAS. Additionally, the correlation patterns among yield-related traits revealed by the pairwise correlation plot establish a phenotypic basis for understanding the genetic patterns and synergistic relationships among rice yield components.

### 3.2. Association Analysis

**Figure 3:**
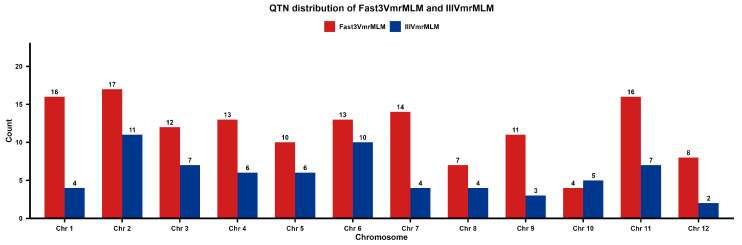
The significant QTNs detected by the Fast3VmrMLM (red) and IIIVmrMLM (blue) method.

GWAS was performed using 4,945,006 SNPs across 12 chromosomes in 529 rice inbred lines. The Fast3VmrMLM method detected 141 significant QTNs associated with all yield-related traits (Figure 3), with *p*-values ranging from 1.8 × 10^−37^ to 3.0 × 10^−6^ and LOD scores from 4.74 to 66.59 (Figure S1). By comparison, the IIIVmrMLM method identified 69 significant QTNs (Figure 3), with *p*-values of 7.0 × 10^−58^ to 6.8 × 10^−7^ and LOD scores of 5.36 to 55.85. Most of the significant QTNs detected by the IIIVmrMLM method were also identified by the Fast3VmrMLM method. Notably, the IIIVmrMLM method failed to detect loci with low phenotypic variance explained (PVE < 1%), whereas Fast3VmrMLM identified substantially more small-effect QTNs. Therefore, all subsequent analyses in this study were conducted based on the results obtained from the Fast3VmrMLM method.

Among all significant QTNs of the Fast3VmrMLM method, 47 loci exhibited low PVE (<1%), indicating small-effect loci that account for approximately 33% of the total QTNs. The cumulative contribution of these small-effect loci was 33.48%. This result highlights the critical role of small-effect QTNs in regulating the genetic variation of target traits, indicating that the genetic architecture of complex traits not only relies on major-effect loci but also that the cumulative effects of several small-effect loci constitute an important genetic basis for trait formation.

**Figure 4:**
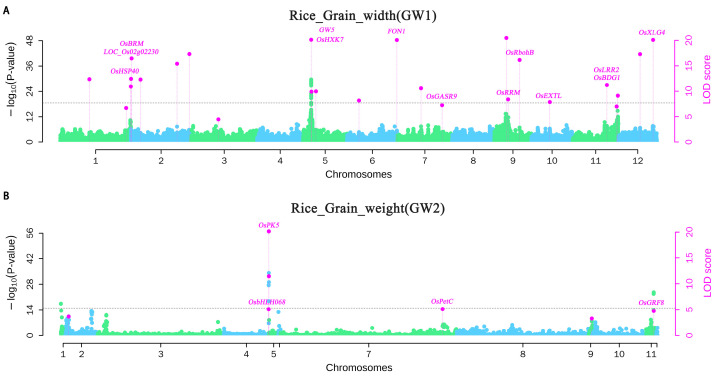
The Manhattan map of two yield-related traits, GW1 (**A**) and GW2 (**B**), by Fast3VmrMLM. The left vertical axis shows log10(*p*-values) of QTNs identified through whole-genome single-marker scanning, while the right axis displays LOD values by likelihood ratio testing. The highlighted text indicates corresponding known genes.

Among them, 23 significant QTNs were detected for GW1, with *p*-values ranging from 1.11 × 10^−27^ to 9.09 × 10^−11^ and LOD scores from 6.97 to 66.59 (Figure 4A). Of these, 16 were small-effect QTNs, accounting for approximately 70% of all QTNs for this trait, and the cumulative contribution of these small-effect loci was 10.78%. Subsequently, the GT, PH, and NEP traits exhibited 22, 22, and 21 QTNs, respectively. For GW2, five significant QTNs were detected (Figure 4B), with *p*-values ranging from 1.80 × 10^−37^ to 3.00 × 10^−6^ and LOD scores from 4.74 to 35.54. Of these, two were small-effect QTNs (40%), with a cumulative PVE of 1.53%. Among these significant QTNs, two QTNs were associated with NEP, NP, and Y traits simultaneously, while five QTNs were associated with both NEP and NP traits. These QTNs appear to exhibit potential polygenic effects.

### 3.3. Known Genes Around Significant QTNs

According to linkage disequilibrium decay (Figure 1A) [25,26], genes located within a 200 kb neighborhood of 141 significant QTNs were detected, and subsequently mapped to previously reported rice yield-related genes. A total of 92 known yield-associated genes were identified around these significant QTNs across all 12 chromosomes, with 21, 13, 14, and 14 known genes corresponding to PH, NEP, GW1, and GT, respectively (Table 2 and Table S1).

Among all detected genes, several were associated with more than one trait; for example, the gene *LOC_Os05g09520*, adjacent to marker vg0505375144 on chromosome 5, was simultaneously associated with GL, GT, and GW1 (Table S1). Additionally, both gene *LOC_Os02g01590* near vg0200420140 on chromosome 2 and gene *LOC_Os09g10960* near vg096031234 were simultaneously associated with NP and NEP (Table 2 and Table S1). This indicates that these genes may exert pleiotropic effects on rice yield-related traits.

Notably, several gene clusters consisting of multiple known yield-related genes were detected around significant QTNs. For instance, four genes, *LOC_Os03g06120*, *LOC_Os03g06139*, *LOC_Os03g06240*, and *LOC_Os03g06410,* located on chromosome 3 (vg0303116237), are all associated with the yield trait. Furthermore, the gene *LOC_Os06g06790* on chromosome 6 was simultaneously associated with HD, NP, and NEP. This gene has been reported to exhibit significant negative correlations with plant height, tiller number, panicle length, and seed setting rate at maturity [30] (Table 2).

### 3.4. Candidate Genes Response to Photoperiod

**Figure 5:**
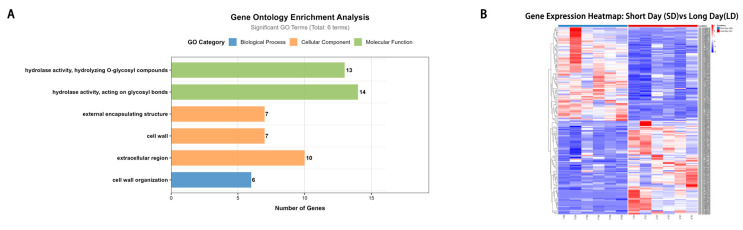
(**A**) Results of gene ontology-based functional enrichment analysis. (**B**) Cluster heatmaps of 182 genes under different photoperiod conditions.

Differential expression analysis was performed to identify genes responsive to photoperiod stress. Among 3855 unreported rice genes, 360 genes were characterized as DEGs under distinct photoperiod treatments. Subsequent GO functional enrichment analysis further revealed that 182 of these 360 DEGs were significantly enriched in six GO terms associated with various biological processes (Figure 5A and Figure S2).

Gene expression levels varied significantly across different photoperiod treatments, with several genes exhibiting divergent expression patterns between SD and LD stress treatments. Specifically, *LOC_Os11g45610*, a gene associated with GL and GW1, located near the SNP vg1127580115 and vg1127655944, displayed a high absolute log_2_FoldChange value of 2.62 at 08:00 under LD treatment, whereas it was only 0.73 at the same time point 08:00 under SD conditions (Figure 5B). Notably, distinct photoperiod-responsive expression patterns were observed between LD and SD treatments. Most genes showed obvious responsive expression profiles under LD conditions (Figure 5B), whereas such responses were absent under SD conditions. For instance, two NP-associated genes, *LOC_Os04g48290* and *LOC_Os04g48350*, which are located near the SNP marker vg0428874691, exhibited absolute log_2_FoldChange values of 3.17 and 3.11 at 12:00 under LD conditions, respectively, while no such significant expression responses were detected under SD conditions. These results clarify the differential photoperiod responsiveness of the identified genes between LD and SD treatments, providing a solid biological basis for characterizing the identified novel photoperiod-responsive genes in rice.

Expression levels of multiple genes also varied at different times under the same photoperiod treatment (Figure 5B). A GT-associated gene, *LOC_Os09g03890*, adjacent to SNP locus vg0901934679, was highly responsive to LD stress, with a high expression level at LD 08:00 at |log_2_FoldChange| = 2.49. However, its expression gradually decreased to 1.44 and 0.30 as light duration extended to LD 12:00 and LD 16:00, respectively. In contrast, *LOC_Os05g09640*, which is associated with GL, GW1, and GT traits, was more sensitive to short-day stress, showing a high expression level at SD 12:00 at |log_2_FoldChange| = 2.72. As light duration increased to LD 16:00 and LD 20:00, its expression progressively declined to 0.66 and 0.01, respectively (Figure 5B).

Among the 182 candidate genes identified via tissue-specific expression analysis, 62 showed significant homology to *Arabidopsis thaliana* genes following sequence alignment, with detailed information on these orthologous gene pairs provided in Table S2. To further refine our candidate gene set and prioritize high-confidence targets, we performed a screening of these 62 homologous genes and ultimately identified six high-confidence pleiotropic candidate genes: *LOC_Os02g02210*, *LOC_Os05g09640*, *LOC_Os09g12590*, *LOC_Os11g37700*, *LOC_Os11g45720*, and *LOC_Os11g45730*. These genes not only display significant allelic variation but also exhibit strong pleiotropic regulatory potential across multiple yield-related traits, thereby justifying their selection for subsequent functional validation.

### 3.5. Haplotype and Phenotypic Difference Analysis of Candidate Genes

**Figure 6:**
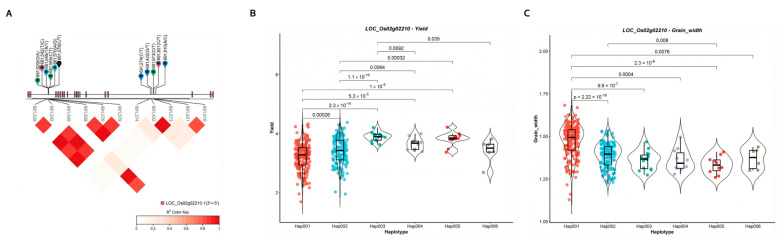
(**A**) Results of haplotype and phenotypic difference analysis for the candidate gene *LOC_Os02g02210*. (**B**) Linkage disequilibrium and haplotype block with 11 SNPs inside for *LOC_Os02g02210*. Comparison of the yield trait among haplotypes 001 (GTTACACCGCCA), 002 (ACTATGTCGCCA), 003 (GTTACACTTTCA), 004 (GCTACGCTGCCA), 005 (GTTACACCTTCA), and 006 (GTTCACCTTTC). (**C**) Comparison of the GW1 trait among haplotypes 001, 002, 003, 004, 005, and 006.

To further validate the association between candidate genes and target traits, haplotype analysis was performed on the candidate gene using SNPs within the gene and its 2 kb upstream and downstream regions. The pleiotropic candidate gene *LOC_Os02g02210* is located near loci vg0200771434 and vg0200654321, which are associated with GW1 and Y (Table 2; Figure 6A). Figure 6B illustrates the LD and haplotype blocks of 11 SNPs. Based on these 11 SNPs (vg0200681529, vg0200681542, vg0200681549, vg0200681569, vg0200681570, vg0200681579, vg0200691274, vg0200691422, vg0200691573, vg0200691621, and vg0200691810), 529 inbred lines were classified into nine haplotypes.

For the yield trait, *t*-tests revealed significant differences between haplotypes 001 and 002 under varying photoperiods (*p* = 2.64 × 10^−4^), as well as between haplotypes 003 and 006 (*p* = 3.55 × 10^−2^) (Figure 6B). For GW1, t-tests indicated statistically significant differences between haplotypes 001 and 002 (*p* = 2.14 × 10^−19^) and between haplotypes 002 and 005 (*p* = 7.80 × 10^−3^) under different photoperiods (Figure 6C). Collectively, these results suggest that the candidate gene *LOC_Os02g02210* may be closely associated with rice yield-related traits.

## 4. Discussion

GWAS is a critical methodology for dissecting the genetic basis of complex traits in crops, and improvements in its statistical models and methodologies are decisive for improving the detection efficacy of genetic loci and deepening our biological understanding. In this study, we employed the Fast3VmrMLM, a compressed mixed model integrating whole-genome scanning and machine learning strategies, to conduct GWAS on rice yield-related traits [20]. Compared with traditional single-locus GWAS methods, this model exhibits significant advantages in detecting QTNs with small genetic effects [17,18]. By analyzing 529 germplasm resources and 4,945,006 SNPs, we identified 141 significant QTNs, among which 92 correspond to previously reported yield-related genes in rice. The detection of these known genes supports the reliability and consistency of the GWAS results. Furthermore, multiple small-effect loci were identified, which may contribute to phenotypic variation through cumulative genetic effects. For example, two yield-associated genes *LOC_Os05g02730* and *LOC_Os05g02770*, near SNP locus vg0501075672 (*r*^2^ = 1.08), regulate the extension of rice growth period, reduction in tiller number, widening of leaves, elongation of panicles, and increase in grains per panicle [31], while significantly decreasing the thousand-grain weight, grain width, and grain thickness [32]. Meanwhile, *LOC_Os04g53990*, a GW2-associated gene adjacent to SNP locus vg0432182557 (*r*^2^ = 0.70), delays rice seed germination [33] and induces phenotypes including dwarf stems, single tillers, small panicles, stunted branches, and fewer small panicles [34]. Although these small-effect genes individually have limited explanatory power for phenotypic variation, their cumulative or interactive effects via pleiotropic networks make non-negligible contributions to the formation of final yield traits [19]. This study highlights that systematic dissection of these small-effect gene networks is essential for comprehensively elucidating the genetic architecture of complex quantitative traits.

In addition to individual significant loci, this study also identified multiple clustered known genes, such as a gene cluster comprising *LOC_Os03g06120*, *LOC_Os03g06139*, *LOC_Os03g06240*, and *LOC_Os03g06410*, located near SNP locus vg0303116237 on chromosome 3, which is associated with yield regulation. Furthermore, haplotype–phenotype association analysis revealed six key candidate genes with pleiotropic potential, especially *LOC_Os02g02210*, which was significantly associated with both GW1 and Y traits. Notably, these genetically identified candidate genes were further validated by differential expression analyses under photoperiod stress. For example, two NP-associated genes, *LOC_Os04g48290* and *LOC_Os04g48350*, exhibited significant expression alterations under LD treatment. The integration of GWAS and transcriptomic data provides complementary evidence for candidate gene prediction. This highlights the utility of multi-omics strategies in gene mining [5].

Environmental factors, particularly photoperiod, are critical regulators of rice growth, development, and yield formation. In this study, photoperiod was treated as a core environmental variable to systematically identify 182 candidate genes with potential responsiveness to photoperiod stress. Analysis revealed that the expression patterns of several genes dynamically changed under different photoperiod conditions or across time points within the same treatment. Through tissue-specific expression analysis, homology analysis, and haplotype–phenotype differential analysis, six pleiotropic candidate genes were confirmed, including *LOC_Os02g02210*, *LOC_Os05g09640*, *LOC_Os09g12590*, *LOC_Os11g37700*, *LOC_Os11g45720*, and *LOC_Os11g45730*. For instance, *LOC_Os05g09640*, which is associated with GL, GW1, and GT, exhibited stronger sensitivity to short-day stress. Meanwhile, *LOC_Os02g02210* was highly responsive to photoperiod and may play important regulatory roles in both grain width and yield-related traits. These findings indicate that photoperiod may influence final yield traits by modulating the expression timing and levels of these genes [18].

In summary, this study employed the Fast3VmrMLM method to systematically dissect the genetic basis of rice yield-related traits. The analysis enabled the detection of QTNs associated with these traits, including several known genes and novel photoperiod-responsive candidates. Through haplotype analysis, differential expression validation, and homologous gene alignment, putative key genes (such as *LOC_Os02g02210*) were identified as potential targets for further study. These findings highlight the importance of small-effect loci and suggest a potential association between photoperiod regulation and the expression of yield-related genes in rice. Overall, this study provides candidate gene resources that will facilitate future investigations into the genetic mechanisms underlying rice yield.

## 5. Conclusions

In this study, we employed Fast3VmrMLM, a compressed mixed-model GWAS integrating whole-genome scanning and machine learning, to detect QTNs and their effects associated with rice yield-related traits. A total of 141 QTNs were identified. Additionally, based on genomic annotation and previous studies, 92 known yield-associated genes were found near these significant QTNs, corresponding to 11, 21, 10, 13, 12, 4, 14, 4, and 14 genes for HD, PH, NP, NEP, Y, GL, GW1, GW2, and GT respectively. Further differential expression and GO functional enrichment analyses identified 182 candidate genes. Through tissue-specific expression analysis, comparison with homologous *Arabidopsis thaliana* genes, and haplotype–phenotype differential analysis, six candidate genes were confirmed, *LOC_Os02g02210*, *LOC_Os05g09640*, *LOC_Os09g12590*, *LOC_Os11g37700*, *LOC_Os11g45720*, and *LOC_Os11g45730*, all of which are pleiotropic genes. Notably, *LOC_Os02g02210* may exert regulatory effects on both grain width and yield traits. These findings provide valuable insights for the mining of genes associated with rice yield.

## Figures and Tables

**Table 1:** Statistical analysis of rice yield-related traits.

Trait	Mean	Max	Min	SD	CV
Heading date	95.73	149.00	45.00	15.31	0.16
Plant height	130.50	206.73	68.40	32.03	0.25
NO. of panicles	13.32	47.20	3.00	5.70	0.43
NO. of effective panicles	13.14	45.80	3.00	5.59	0.43
Yield	31.03	92.66	4.41	13.94	0.45
Grain weight	23.50	41.48	14.01	3.78	0.16
Spikelet length	23.68	33.34	13.68	3.17	0.13
Grain length	8.57	10.97	6.13	0.88	0.10
Grain width	3.12	4.38	1.96	0.39	0.12
Grain thickness	2.00	2.45	1.58	0.14	0.07

**Table 2:** The detected known pleiotropic genes of yield-related traits.

Trait	Gene	Chr	SNP (bp)	*p* Value	LOD	*r*^2^ (%)
NP/NEP	*LOC_Os02g01590*	2	420140	2.72 × 10^−11^	9.64	1.79
GW1/Y	*LOC_Os02g02230*	2	654321	3.24 × 10^−18^	16.45	0.80
GT/GW1	*LOC_Os05g09500*	5	5375144	3.93 × 10^−33^	31.23	4.57
GL/GT/GW1	*LOC_Os05g09520*	5	5375144	3.93 × 10^−33^	31.23	4.57
HD/NP/NEP	*LOC_Os06g06790*	6	3239372	3.59 × 10^−31^	29.28	2.37
HD/NP/NEP	*LOC_Os06g06900*	6	3239372	3.59 × 10^−31^	29.28	2.37
NP/NEP	*LOC_Os09g10960*	9	6031234	4.90 × 10^−15^	13.31	1.30

## Data Availability

The original contributions presented in this study are included in the article/Supplementary Material. Further inquiries can be directed to the corresponding author.

## Supplementary Materials

The following supporting information can be downloaded at: https://www.mdpi.com/article/10.3390/plants15060875/s1, Table S1: Known genes detected by the Fast3VmrMLM method for rice. Table S2: Orthologous information of candidate genes with higher tissue expression. Table S3: The germplasm information of 529 Asian cultivated rice. Figure S1: The Manhattan map of 8 rice traits by using the Fast3VmrMLM method. The left vertical axis shows log10(*p*-values) of QTNs identified through whole-genome single-marker scanning, while the right axis displays LOD values identified by likelihood ratio testing. The highlighted text indicates corresponding known genes. Figure S2: The graph of GO terms in biological process by enrichment analysis.

## Abbreviations

The following abbreviations are used in this manuscript: GWAS: genome-wide association study; SNP: single nucleotide polymorphism; QTN: quantitative trait nucleotides; GO: gene ontology; EMMA: efficient mixed-model association expedited; GEMMA: genome-wide efficient mixed-model association; HD: heading date; PH: plant height; NP: number of panicles; NEP: number of effective panicles; Y: yield; SL: spikelet length; GL: grain length; GW1: grain width; GW2: grain weight; GT: grain thickness; CV: coefficient of variation; MAF: minor allele frequency; GEO: gene expression omnibus; DEG: differentially expressed gene; SD: short-day; LD: long-day.
