# Supplementary material for: Genome-Wide Association Study of Yield-Related Traits and Photoperiod Response in Rice
Source: Plants (Basel). 2026 Mar 12;15(6):875. doi: 10.3390/plants15060875 (PMC13029942; doi:10.3390/plants15060875)
Supplement: Supplementary file 1 [file plants-15-00875-s001.zip › plants-4138116-supplementary.pdf]

Genome-Wide Association Study of Yield-Related Traits and Photoperiod Response in Rice

Supplementary material

Ziming Zang [1,2,†], Chang Liu [2,†], Zhaoqin Wang [2], Cheng Fan [2] and Juncong Chen [1,*]

1 School of Finance, Nanjing Agricultural University, Nanjing 210095, China
2 College of Sciences, Nanjing Agricultural University, Nanjing 210095, China
* Correspondence: jcchen@njau.edu.cn
† These authors contributed equally to this work.

Table S1 Known gene detected by Fast3VmrMLM method of rice.

Trait	Marker	bp	Gene	Gene symbol	Trait	Marker	bp	Gene	Gene symbol
PH	vg0124440483	24440483	LOC_Os01g42950	OsSCYL2	NEP	vg0110539594	10539594	LOC_Os01g18800	OsCIPK1
	vg0132162630	32162630	LOC_Os01g56000	OsEOG1		vg0200420140	420140	LOC_Os02g01590	sgs1
	vg0135137192	35137192	LOC_Os01g60810	OsOFP6		vg0219760576	19760576	LOC_Os02g33110	OsCIN1
			LOC_Os01g60900	OsEPFL9		vg0423185294	23185294	LOC_Os04g39020	OsBADH1
	vg0135652366	35652366	LOC_Os01g61500	OsBAG4				LOC_Os04g39160	OsRDR2
	vg0303427295	3427295	LOC_Os03g06700	OsKCS11		vg0431533472	31533472	LOC_Os04g52860	FLR8
			LOC_Os03g06705	OsCUT3				LOC_Os04g52920	GSD1
	vg0334080119	34080119	LOC_Os03g59790	OsEFH1				LOC_Os04g52960	OsNUC1
			LOC_Os03g60080	SNAC1		vg0601663265	1663265	LOC_Os06g04010	OsGBP1
	vg0334424902	34424902	LOC_Os03g60570	OsZFP15		vg0603239372	3239372	LOC_Os06g06790	OsPDIL1-5
			LOC_Os03g60720	OsEXPA7				LOC_Os06g06900	SD6
	vg0432681182	32681182	LOC_Os04g54900	OsILI1		vg0617621990	17621990	LOC_Os06g30370	OsMFT1
	vg0626674715	26674715	LOC_Os06g44300	OsGL1-3		vg0906031234	6031234	LOC_Os09g10960	OsMED14_2
			LOC_Os06g44310	OsTET10	Y	vg0200771434	771434	LOC_Os02g02230	LOC_Os02g02230
			LOC_Os06g44320	OsbHLH158				LOC_Os02g02400	OsCAT2
	vg0627447078	27447078	LOC_Os06g45460	APO1		vg0302773210	2773210	LOC_Os03g05730	OsCDC48
	vg0630182572	30182572	LOC_Os06g49740	OsSYF2		vg0303116237	3116237	LOC_Os03g06120	OsMCA1
	vg0804261000	4261000	LOC_Os08g07740	DTH8				LOC_Os03g06139	OsABCG22
			LOC_Os08g07760	OsSERK1				LOC_Os03g06240	YTH03
	vg0914221572	14221572	LOC_Os09g23820	CYP735A4				LOC_Os03g06410	OsMAPKKK1
	vg1123558684	23558684	LOC_Os11g39670	TSCD11		vg0501075672	1075672	LOC_Os05g02730	GLR1
GT	vg0200145873	145873	LOC_Os02g01360	qHd2-1				LOC_Os05g02770	OsGPRP3
	vg0203453596	3453596	LOC_Os02g06910	OsARF6		vg1017472201	17472201	LOC_Os10g33230	RBP-J
	vg0316961797	16961797	LOC_Os03g29614	SMG3				LOC_Os10g33310	OsKRP4
	vg0335915711	35915711	LOC_Os03g63540	OsGP1		vg1104999384	4999384	LOC_Os11g09280	esp2
			LOC_Os03g63600	OsTET5	HD	vg0107209574	7209574	LOC_Os01g12890	OsEMF1
			LOC_Os03g63620	OsTET6		vg0132459999	32459999	LOC_Os01g56380	TYDC
	vg0432864652	32864652	LOC_Os04g55159	OsAAI1		vg0301376034	1376034	LOC_Os03g03164	OsKNAT7
	vg0504810769	4810769	LOC_Os05g08810	PI3K		vg0603243535	3243535	LOC_Os06g06790	OsPDIL1-5
	vg0505379423	5379423	LOC_Os05g09500	OsHXK7				LOC_Os06g06900	SD6
			LOC_Os05g09520	GW5		vg1118954207	18954207	LOC_Os11g32110	OsARF23
	vg0523855122	23855122	LOC_Os05g40770	OsRPK1				LOC_Os11g32160	SSG7

Trait	Marker	bp	Gene	Gene symbol	Trait	Marker	bp	Gene	Gene symbol
	vg0718958684	18958684	*LOC_Os07g32040*	*NY2*				*LOC_Os11g32240*	*OsCYP51G1*
	vg0821742266	21742266	*LOC_Os08g34550*	*WLG*		vg1120135588	20135588	*LOC_Os11g34300*	*OsMRG702*
	vg1123281267	23281267	*LOC_Os11g39000*	*ILI2*				*LOC_Os11g34460*	*OsFKF1*
GW1	vg0200066718	66718	*LOC_Os02g01030*	*OsHSP40*		vg1219847331	19847331	*LOC_Os12g32980*	*SAB23*
	vg0200654321	654321	*LOC_Os02g02230*	*LOC_Os02g02230*	NP	vg0200420140	420140	*LOC_Os02g01590*	*sgs1*
			LOC_Os02g02290	*OsBRM*		vg0234054213	34054213	*LOC_Os02g55440*	*OsTMN11*
	vg0505375144	5375144	*LOC_Os05g09500*	*OsHXK7*		vg0428874691	28874691	*LOC_Os04g48460*	*CYP704A3*
			LOC_Os05g09520	*GW5*				*LOC_Os04g48490*	*DEAP1*
	vg0630519365	30519365	*LOC_Os06g50340*	*FON1*		vg0603239372	3239372	*LOC_Os06g06750*	*OsMADS5*
	vg0724114396	24114396	*LOC_Os07g40240*	*OsGASR9*				*LOC_Os06g06790*	*OsPDIL1-5*
	vg0907281787	7281787	*LOC_Os09g12730*	*OsRRM*				*LOC_Os06g06900*	*SD6*
	vg0916232414	16232414	*LOC_Os09g26660*	*OsRbohB*		vg0803245470	3245470	*LOC_Os08g05910*	*OsNRT1.1A*
	vg1011745770	11745770	*LOC_Os10g22590*	*OsEXTL*				*LOC_Os08g06100*	*OsCOMT*
	vg1118478534	18478534	*LOC_Os11g31530*	*OsBDG1*		vg0906031234	6031234	*LOC_Os09g10960*	*OsMED14_2*
			LOC_Os11g31540	*OsLRR2*	GW2	vg0432182557	32182557	*LOC_Os04g53990*	*OsbHLH068*
	vg1224917812	24917812	*LOC_Os12g40190*	*OsXLG4*		vg0434700310	34700310	*LOC_Os04g58110*	*OsPK5*
GL	vg0216615524	16615524	*LOC_Os02g28074*	*OsXRNL*		vg0722156245	22156245	*LOC_Os07g37030*	*OsPetC*
	vg0314076882	14076882	*LOC_Os03g24600*	*OsMSRB5*		vg1120507010	20507010	*LOC_Os11g35030*	*OsGRF8*
	vg0505358756	5358756	*LOC_Os05g09520*	*GW5*					
	vg1204516417	4516417	*LOC_Os12g08780*	*OsYUC11*					

Table S2 Orthologous information of candidate genes with higher tissue expression

Trait	Gene	Maker	Arabidopsis Orthologous gene	Putative function
GL	*LOC_Os02g10990*	vg0205876935	AT3G15620	DNA photolyase family protein
GL	*LOC_Os02g11020*	vg0205876935	AT2G26710	Cytochrome P450 superfamily protein
GL/GT/GW1	*LOC_Os05g09640*	vg0505358756	AT2G19780	Leucine-rich repeat (LRR) family protein
GL	*LOC_Os07g01770*	vg0700409164	AT2G01540	Calcium-dependent lipid-binding (CaLB domain) family protein
GL/GW1	*LOC_Os11g45720*	vg1127580115	AT5G61680	Pectin lyase-like superfamily protein
GL/GW1	*LOC_Os11g45730*	vg1127580115	AT5G07410	Pectin lyase-like superfamily protein
GT	*LOC_Os01g55549*	vg0131952193	AT1G79840	HD-ZIP IV family of homeobox-leucine zipper protein with lipid-binding START domain-containing protein
GT	*LOC_Os01g55590*	vg0131952193	AT3G16170	AMP-dependent synthetase and ligase family protein
GT	*LOC_Os03g58110*	vg0333127012	AT3G22800	Leucine-rich repeat (LRR) family protein
GT	*LOC_Os03g58320*	vg0333127012	AT4G02610	Aldolase-type TIM barrel family protein
GT	*LOC_Os03g63580*	vg0335915711	AT3G03530	non-specific phospholipase C4
GT	*LOC_Os05g26926*	vg0515696564	AT4G39960	Molecular chaperone Hsp40/DnaJ family protein
GT	*LOC_Os05g27010*	vg0515696564	AT2G40460	Major facilitator superfamily protein
GT	*LOC_Os05g40740*	vg0523855122	AT3G13400	SKU5 similar 13
GT	*LOC_Os07g31840*	vg0718958684	AT1G28340	receptor like protein 4
GT	*LOC_Os08g14450*	vg0808722420	AT2G36990	RNApolymerase sigma-subunit F
GT	*LOC_Os09g03890*	vg0901934679	AT4G32070	Octicosapeptide/Phox/Bem1p (PB1) domain-containing protein / tetratricopeptide repeat (TPR)-containing protein
GW1	*LOC_Os01g74450*	vg0143210484	AT4G01470	tonoplast intrinsic protein 1;3
GW1/Y	*LOC_Os02g02210*	vg0200654321	AT3G22200	Pyridoxal phosphate (PLP)-dependent transferases superfamily protein
GW1	*LOC_Os02g56920*	vg0234925260	AT1G02190	Fatty acid hydroxylase superfamily
GW1	*LOC_Os02g57120*	vg0234925260	AT2G20190	CLIP-associated protein
GW1	*LOC_Os06g50400*	vg0630519365	AT1G20190	expansin 11
GW1/PH	*LOC_Os09g12590*	vg0907281787	AT1G11260	sugar transporter 1
GW1	*LOC_Os12g40130*	vg1224917812	AT1G27450	adenine phosphoribosyl transferase 1
GW2	*LOC_Os04g58200*	vg0434700310/vg 0434739892	AT5G54190	protochlorophyllide oxidoreductase A
GW2	*LOC_Os07g37100*	vg0722156245	AT4G05110	equilibrative nucleoside transporter 6
HD	*LOC_Os01g34920*	vg0119203457	AT1G31740	beta-galactosidase 15
HD	*LOC_Os01g56420*	vg0132459999	AT5G59030	copper transporter 1
HD	*LOC_Os03g03370*	vg0301376034	AT4G25700	beta-hydroxylase 1
HD	*LOC_Os08g40180*	vg0825395199	AT1G76490	hydroxy methylglutaryl CoA reductase 1
HD	*LOC_Os11g32030*	vg1118954207	AT3G26760	NAD(P)-binding Rossmann-fold superfamily protein

Trait	Gene	Maker	Arabidopsis Orthologous gene	Putative function
HD	*LOC_Os12g32760*	vg1219847331	AT3G18830	polyol/monosaccharide transporter 5
NEP	*LOC_Os06g03990*	vg0601663265	AT5G51690	1-amino-cyclopropane-1-carboxylate synthase 12
NEP	*LOC_Os06g04070*	vg0601663265	AT4G34710	arginine decarboxylase 2
NEP	*LOC_Os06g04150*	vg0601663265	AT4G25080	magnesium-protoporphyrin IX methyltransferase
NEP	*LOC_Os07g41580*	vg0724993619	AT4G14540	nuclear factor Y, subunit B3
NEP/NP/Y	*LOC_Os11g37700*	vg1122281239	AT2G36380	pleiotropic drug resistance 6
NP	*LOC_Os04g48290*	vg0428874691	AT4G23030	MATE efflux family protein
NP	*LOC_Os04g48400*	vg0428874691	AT1G12570	Glucose-methanol-choline (GMC) oxidoreductase family protein
NP	*LOC_Os04g48530*	vg0428874691	AT1G12480	C4-dicarboxylate transporter/malic acid transport protein
PH	*LOC_Os01g42880*	vg0124440483	AT3G62160	HXXXD-type acyl-transferase family protein
PH	*LOC_Os01g42900*	vg0124440483	AT1G53270	ABC-2 type transporter family protein
PH	*LOC_Os01g55690*	vg0132162630	AT5G44120	RmlC-like cupins superfamily protein
PH	*LOC_Os01g55950*	vg0132162630	AT4G37560	Acetamidase/Formamidase family protein
PH	*LOC_Os01g60770*	vg0135137192	AT2G40610	expansin A8
PH	*LOC_Os03g06760*	vg0303427295	AT3G56640	exocyst complex component sec15A
PH	*LOC_Os03g06940*	vg0303427295	AT4G36360	beta-galactosidase 3
PH	*LOC_Os03g60000*	vg0334080119	AT4G28510	prohibitin 1
PH	*LOC_Os03g60380*	vg0334424902	AT5G58490	NAD(P)-binding Rossmann-fold superfamily protein
PH	*LOC_Os04g54810*	vg0432681182	AT5G64570	beta-D-xylosidase 4
PH	*LOC_Os04g54930*	vg0432681182	AT3G28860	ATP binding cassette subfamily B19
PH	*LOC_Os06g44250*	vg0626674715	AT4G30850	heptahelical transmembrane protein2
PH	*LOC_Os06g45410*	vg0627447078	AT1G79430	Homeodomain-like superfamily protein
PH	*LOC_Os09g08130*	vg0904293220	AT2G04400	Aldolase-type TIM barrel family protein
PH	*LOC_Os11g42450*	vg1125588062	AT1G03440	Leucine-rich repeat (LRR) family protein
Y	*LOC_Os01g04640*	vg0102101327	AT3G18260	Reticulon family protein
Y	*LOC_Os01g04720*	vg0102101327	AT1G25570	Di-glucose binding protein with Leucine-rich repeat domain-containing protein
Y	*LOC_Os02g02310*	vg0200771434	AT1G75500	Walls Are Thin 1
Y	*LOC_Os02g53820*	vg0233050045	AT3G43860	glycosyl hydrolase 9A4
Y	*LOC_Os02g54060*	vg0233050045	AT5G20720	chaperonin 20
Y	*LOC_Os09g35940*	vg0920723867	AT3G61880	cytochrome p450 78a9
Y	*LOC_Os09g36060*	vg0920723867	AT4G39010	glycosyl hydrolase 9B18

Table S3 The germplasm information of 529 Asian cultivated rice.

Cultivar Name	Cultivar ID	Subpopulation	Location	Accession
Sadu-cho	C001	Indica I	Philippines	SRR1239601
Dom_Sufid	C002	VI/Aromatic	Philippines	SRR1239602
Gerdeh	C003	Japonica Intermediate	Iran	SRR1239603
Li-Jiang-Xin-Tuan-Hei-Gu_	C004	Temperate Japonica	China	SRR1239604
AZUCENA	C005	Tropical Japonica	Philippines	SRR1239605
Shan-Huang_Zhan-2-1_	C006	Indica Intermediate	China	SRR1239606
Shan-Huang_Zhan-2-2_	C007	Indica Intermediate	India	SRR1239607
Swarna	C008	Indica Intermediate	Guinea	SRR1239608
MOROBEREKAN	C009	Indica Intermediate	United States	SRR1239609
CYPRESS	C010	Tropical Japonica	Philippines	SRR1239610
MILYANG_23	C011	Indica II	Taiwan	SRR1239611
Tainong67	C012	Temperate Japonica	Philippines	SRR1239612
N_22	C013	Aus	Philippines	SRR1239613
M202	C014	Japonica Intermediate	India	SRR1239614
Dular	C015	Aus	Heilongjiang	SRR1239615
Laoguangtou83	C016	Temperate Japonica	Heilongjiang	SRR1239616
AnnongwangengB-1	C017	Temperate Japonica	Anhui	SRR1239617
AnnongwangengB-2	C018	Temperate Japonica	Zhejiang	SRR1239618
Aijiaonante	C019	Indica I	Guangdong	SRR1239619
Guangluai-4-1	C020	Indica I	Hunan	SRR1239620
xiangaizao10	C021	Indica I	Unknown	SRR1239621
JinnanteB	C022	Indica I	Hunan	SRR1239622
Funingzipi	C023	Temperate Japonica	Hebei	SRR1239623
zhenshan97B-1	C024	Indica I	Jiangxi	SRR1239624
Qingsiai16B	C025	Indica Intermediate	Guangdong	SRR1239625
Weiguo	C026	Temperate Japonica	Liaoning	SRR1239626
Dianrui409B	C027	Indica Intermediate	Yunnan	SRR1239627
Liaogeng287	C028	Temperate Japonica	Liaoning	SRR1239628
Huhui628	C029	Japonica Intermediate	Unknown	SRR1239629
88B-1	C030	Indica Intermediate	Jiangsu	SRR1239630
88B-2	C031	Indica Intermediate	Hunan	SRR1239631
Longhuamaohu	C032	Temperate Japonica	Hebei	SRR1239632
Lucaihao	C033	Indica I	Fujian	SRR1239633
Zhonglouyihao1	C034	Temperate Japonica	Shanxi	SRR1239634
Yelicanghua	C035	Temperate Japonica	Hebei	SRR1239635
Shufeng101	C036	Indica Intermediate	Sichuan	SRR1239636
Chengduai3hao	C037	Indica I	Sichuan	SRR1239637
Sankecui	C038	Indica Intermediate	Hunan	SRR1239638
Gongju73	C039	Indica I	Yunnan	SRR1239639
Jiabala	C040	Indica I	Xizang	SRR1239640
Taishannuo	C041	Indica I	Guangdong	SRR1239641
Guichao2hao	C042	Indica Intermediate	Guangdong	SRR1239642
Huke3hao	C043	Indica I	Shanghai	SRR1239643
Teqingxuanhui	C044	Indica Intermediate	Unknown	SRR1239644
Huangsiguizhan	C045	Indica Intermediate	Guangdong	SRR1239645
Xiangwanxian3hao	C046	Indica Intermediate	Hunan	SRR1239646
Hanmadao-2	C047	Indica I	Taiwan	SRR1239647
Zaoshunonghu6	C048	Temperate Japonica	Hunan	SRR1239648
Jinyou1hao	C049	Indica Intermediate	Fujian	SRR1239649
Chengnongshuijing	C050	Indica II	Sichuan	SRR1239650
PeiC122	C051	Intermediate	Unknown	SRR1239651
Guihuahuang	C052	Temperate Japonica	Jiangsu	SRR1239652
Momi	C053	Indica Intermediate	Guangxi	SRR1239653
Xiushui115	C054	Temperate Japonica	Zhejiang	SRR1239654
Sanbaili	C055	Indica I	Hunan	SRR1239655
Jindao1hao	C056	Temperate Japonica	Shanxi	SRR1239656
Dandongludao	C057	Japonica Intermediate	Liaoning; China	SRR1239657
Liusha1hao	C058	Indica I	Guangxi; China	SRR1239658
Bawangbian1	C059	Indica I	Hubei	SRR1239659
Dongtingwanxian	C060	Indica I	Hunan	SRR1239660
Yangdao2hao	C061	Indica II	Jiangsu	SRR1239661
WH62	C062	Indica I	Henan	SRR1239662
Geng87-304	C063	Temperate Japonica	Hunan	SRR1239663
Sugeng2hao	C064	Temperate Japonica	Jiangsu	SRR1239664
Hongwan1hao	C065	Indica Intermediate	Fujian	SRR1239665
Vary_Tarva_Osla	W063	Japonica Intermediate	Portugal	SRR1239866
CSORNUJ	W064	Temperate Japonica	Hungary	SRR1239867
R_67	W065	Tropical Japonica	Senegal	SRR1239868
IR_238	W066	Indica Intermediate	Philippines	SRR1239869
Mitak	W067	Tropical Japonica	Indonesia	SRR1239870
Gazan	W068	Temperate Japonica	Afghanistan	SRR1239871
99216	W069	Aus	India	SRR1239872
Shui_Ya_Jien	W070	Indica Intermediate	Hong Kong	SRR1239873
AKP_4	W071	Indica Intermediate	India	SRR1239874
SORNAVARI	W072	Aus	Mali	SRR1239875
IR_2061-214-2-3	W073	Indica II	Philippines	SRR1239876
TAINUNG_45	W074	Indica Intermediate	Taiwan	SRR1239877
Sapundali_Local	W075	Indica Intermediate	India	SRR1239878
Tauli	W076	Aus	Nepal	SRR1239879
79	W077	VI/Aromatic	Guyana	SRR1239880
B805D-MR-16-8-3	W078	Indica Intermediate	Indonesia	SRR1239881
A_152	W079	Intermediate	Bangladesh	SRR1239882
UZ_ROS_59	W080	Indica I	Uzbekistan	SRR1239883
Gasym_Hany	W081	VI/Aromatic	Azerbaijan	SRR1239884
Celiaj	W082	Temperate Japonica	Azerbaijan	SRR1239885
CNTLR80076-44-1-1-1	W083	Indica Intermediate	Thailand	SRR1239886
IR_58614-B-B-8-2	W084	Indica Intermediate	Philippines	SRR1239887
KECHENGNUO_NO._4	W085	Indica Intermediate	China	SRR1239888
4484	W086	Indica II	China	SRR1239889
YOU-I_B	W087	Indica I	China	SRR1239890
CHUNJIANGZAO_NO._1	W088	Temperate Japonica	China	SRR1239891
Egyptian_Wild_Type	W089	Indica III	Turkey	SRR1239892
C.B._II	W090	Aus	Japan	SRR1239893
Gallawa	W091	Aus	Sri Lanka	SRR1239894
Karayal	W092	Aus	Sri Lanka	SRR1239895
Srav_Prapay	W093	Indica III	Cambodia	SRR1239896
Nang_Bang_Bentre	W094	Aus	Vietnam	SRR1239897
DJ_24	W095	Aus	Bangladesh	SRR1239898
DJ_102	W096	Aus	Bangladesh	SRR1239899
Santhi_990	W097	Intermediate	Pakistan	SRR1239900
UZ_ROS_7-13	W098	Aus	Uzbekistan	SRR1239901
SL_22-620	W099	Aus	Sierra Leone	SRR1239902
Spin_Mere	W100	Aus	Afghanistan	SRR1239903
AMANE	W101	Indica Intermediate	Sri Lanka	SRR1239904
Padi_Tarab_Arab	W102	Tropical Japonica	Malaysia	SRR1239905
P_35	W103	Aus	India	SRR1239906
CAROLINO_164	W104	Aus	Chad	SRR1239907
HKG_98	W105	Aus	Mali	SRR1239908
Daudzai_Field_Mix	W106	Aus	Pakistan	SRR1239909
JP_5	W107	Indica Intermediate	Pakistan	SRR1239910
Hi_Muke	W108	Aus	Kazakhstan	SRR1239911
WIR_911	W109	Temperate Japonica	Russian	SRR1239912
Lua_Chua_Chan	W110	Tropical Japonica	Vietnam	SRR1239913
Sereno	W111	Indica III	Jamaica	SRR1239914
ARC_10633	W112	Indica III	India	SRR1239915
Simpor	W113	Tropical Japonica	Brunei	SRR1239916
Heo_Trang	W114	Indica III	Vietnam	SRR1239917
THAVALU	W115	Aus	Sri Lanka	SRR1239918
WC_10253	W116	Tropical Japonica	Unknown	SRR1239919
KRASNODARSKIJ_3352	W117	Temperate Japonica	Russian	SRR1239920
EMBRAPA_1200	W118	Tropical Japonica	Brazil	SRR1239921
WAB462-10-3-1	W119	Tropical Japonica	Cote D'Ivoire	SRR1239922
Bombilla	W120	Temperate Japonica	Spain	SRR1239923
TCHAMPA	W121	Aus	Iran	SRR1239924
BHIM_DHAN	W122	Japonica Intermediate	Nepal	SRR1239925
WC_3532	W123	Tropical Japonica	Peru	SRR1239926
Kin_Shan_Zim	W124	Indica I	China	SRR1239927
Yong_Chal_Byo	W125	Intermediate	South Korea	SRR1239928
Pan_Ju	W126	Indica I	China	SRR1239929
Buphopa	W127	Japonica Intermediate	Myanmar	SRR1239930

Cultivar Name	Cultivar ID	Subpopulation	Location	Accession
Liushizao	C066	Indica I	Sichuan	SRR1239666
Muxiqiu	C067	Temperate Japonica	Jiangsu	SRR1239667
Taizhongxianxuan2	C068	Indica II	Taiwan	SRR1239668
Dangyu5hao	C069	Indica II	Anhui	SRR1239669
Youmangzaogeng	C070	Temperate Japonica	Jiangsu	SRR1239670
Laohuzhong	C071	Temperate Japonica	Shanghai	SRR1239671
Nantehao	C072	Indica I	Jiangxi	SRR1239672
Esiniu	C073	Indica I	Guangdong	SRR1239673
cunsanli	C074	Temperate Japonica	Jiangsu	SRR1239674
Huangkezaonian	C075	Temperate Japonica	Jiangsu	SRR1239675
Benbanggu-1	C076	Indica I	Sichuan	SRR1239676
Benbanggu-2	C077	Indica I	Yunnan	SRR1239677
Qitoubaigu	C078	Indica Intermediate	Yunnan	SRR1239678
Muguanuo-1	C079	Temperate Japonica	Hunan	SRR1239679
Hanmadao-1	C080	Indica I	Henan	SRR1239680
Heidu4	C081	Indica I	Guangdong	SRR1239681
Gaoyangdiandao	C082	Temperate Japonica	Hebei	SRR1239682
chikenuo	C083	Temperate Japonica	Fujian	SRR1239683
Haobuka	C084	Japonica Intermediate	Yunnan	SRR1239684
Shanjiugu	C085	Japonica Intermediate	Sichuan	SRR1239685
Fanhaopi	C086	Aus	Yunnan	SRR1239686
Lixingeng	C087	Temperate Japonica	Sichuan	SRR1239687
Nangaogu-2	C088	Indica I	Jiangsu	SRR1239688
Tieganwu	C089	Temperate Japonica	Zhejiang	SRR1239689
Sanlicun	C090	Indica Intermediate	Shanxi	SRR1239690
Meihuanuo-1	C091	Indica I	Sichuan	SRR1239691
Meihuanuo-2	C092	Indica I	Yunnan	SRR1239692
Xiangnuo-2	C093	Temperate Japonica	Guizhou	SRR1239693
Menjiagao1	C094	Indica Intermediate	Hainan	SRR1239694
Xiaohonggu	C095	Indica I	Yunnan	SRR1239695
Jinxibai-1	C096	Indica Intermediate	Jiangxi	SRR1239696
Jinbaoyin	C097	Indica Intermediate	Fujian	SRR1239697
Hongainuo	C098	Indica Intermediate	Guangxi	SRR1239698
WH099	C099	Indica Intermediate	Guangxi	SRR1239699
WH100	C100	Indica Intermediate	Hubei	SRR1239700
Babaili	C101	Temperate Japonica	Yunnan	SRR1239701
Qiyuexian	C102	Indica Intermediate	Guangxi	SRR1239702
Yuyannuo-1	C103	Temperate Japonica	Yunnan	SRR1239703
Mamagu-1	C104	Indica I	Sichuan	SRR1239704
WH105	C105	Indica Intermediate	Yunnan	SRR1239705
Hongkezhenuo	C106	Temperate Japonica	Guizhou	SRR1239706
Mowanggunei-2	C107	Indica III	Yunnan	SRR1239707
Mamagu-2	C108	Indica I	Sichuan	SRR1239708
WH109	C109	Indica I	Yunnan	SRR1239709
Xianggu	C110	Indica I	Yunnan	SRR1239710
Ximaxian	C111	Temperate Japonica	Yunnan	SRR1239711
Jinnante43B	C112	Indica I	Hunan	SRR1239712
Xiangzaoxian7hao	C113	Indica Intermediate	Hunan	SRR1239713
80B	C114	Indica Intermediate	Hunan	SRR1239714
WH115	C115	Indica II	Hunan	SRR1239715
LimingB	C116	Temperate Japonica	Liaoning	SRR1239716
Jiangnongzao1hao	C117	Indica Intermediate	Jiangxi	SRR1239717
Gu154	C118	Indica II	Unknown	SRR1239718
Ninghui21	C119	Temperate Japonica	Unknown	SRR1239719
Shuiyuan300li	C120	Temperate Japonica	Tianjin	SRR1239720
XiangaiB	C121	Indica Intermediate	Jiangxi	SRR1239721
Aituogu151	C122	Indica I	Sichuan	SRR1239722
Taidongludao	C123	Tropical Japonica	Taiwan	SRR1239723
menjiading2	C124	Indica Intermediate	Hainan	SRR1239724
Jiefangxian	C125	Indica I	Jiangxi	SRR1239725
Baikehualuo	C127	Indica I	Guangdong	SRR1239727
Liuyezhan	C128	Indica I	Hubei	SRR1239728
Aihechi	C129	Indica Intermediate	Jiangxi	SRR1239729
Xiangnuo-1	C130	Temperate Japonica	Guizhou	SRR1239730
Xuanenchangtan	C131	Indica I	Hubei	SRR1239731
Jinzhinuo	C132	Indica Intermediate	Yunnan	SRR1239732
Muguanuo-2	C133	Temperate Japonica	Shanxi	SRR1239733
Wuzidui	C134	Temperate Japonica	Yunnan	SRR1239734

Cultivar Name	Cultivar ID	Subpopulation	Location	Accession
TAINO_38	W128	Intermediate	Taiwan	SRR1239931
2	W129	VI/Aromatic	Afghanistan	SRR1239932
LUSITANO	W130	Temperate Japonica	Portugal	SRR1239933
IARI_6621	W131	Aus	India	SRR1239934
Hsin_Hsing_Pai_Ku	W132	Indica I	Taiwan	SRR1239935
TD_70	W133	Indica Intermediate	Thailand	SRR1239936
Dara	W134	Aus	Indonesia	SRR1239937
Shimla_Early	W135	Indica Intermediate	Iraq	SRR1239938
LA_PLATA_GENA_F.A.	W136	VI/Aromatic	Argentina	SRR1239939
CM1;_HAIPONG	W137	Indica Intermediate	Vietnam	SRR1239940
4595	W138	Indica I	China	SRR1239941
A_5	W139	Temperate Japonica	Japan	SRR1239942
Ittikulama	W140	Aus	Sri Lanka	SRR1239943
DNJ_179	W141	Aus	Bangladesh	SRR1239944
DNJ_121	W142	Aus	Bangladesh	SRR1239945
Jyanak	W143	Japonica Intermediate	Bhutan	SRR1239946
Ao_Chiu_2_Hao	W144	Indica I	China	SRR1239947
GUYANE_1	W145	Indica Intermediate	Chad	SRR1239948
10340	W146	Indica I	Italy	SRR1239949
BR11	W147	Indica Intermediate	Bangladesh	SRR1239950
BR24	W148	Indica Intermediate	India	SRR1239951
93072	W149	Indica Intermediate	China	SRR1239952
108S	W150	Indica II	Unknown	SRR1239953
C418	W151	Indica II	China	SRR1239954
CDR22	W152	Indica II	China	SRR1239955
Chenghui448	W153	Indica II	Sichuan	SRR1239956
Fengaizhan	W154	Indica II	Guangdong	SRR1239957
Gang46B	W155	Indica Intermediate	China	SRR1239958
Gumei2hao	W156	Indica I	Sichuan	SRR1239959
Huajingxian74	W157	Indica Intermediate	Guangdong	SRR1239960
M3122	W158	Intermediate	China	SRR1239961
R644	W159	Indica II	China	SRR1239962
Shennong265	W160	Temperate Japonica	Unknown	SRR1239963
Y134	W161	Indica II	China	SRR1239964
Yuanjing7	W162	Temperate Japonica	China	SRR1239965
Yuexiangzhan	W163	Indica Intermediate	Guangdong	SRR1239966
Zaoxian14	W164	Indica Intermediate	Unknown	SRR1239967
Zhong413	W165	Indica Intermediate	Zhejiang	SRR1239968
Zhongyouzao81	W166	Indica Intermediate	Zhejiang	SRR1239969
Zihui100	W167	Indica Intermediate	Anhui	SRR1239970
Bg90-2	W168	Indica II	Brazil	SRR1239971
IR72	W169	Indica II	Unknown	SRR1239972
PR106	W170	Indica II	Pakistan	SRR1239973
TKM9	W171	Indica Intermediate	Philippines	SRR1239974
Amo13_(Sana)	W172	Indica II	Unknown	SRR1239975
Khazar	W173	Tropical Japonica	Iran	SRR1239976
Gayabyeo	W174	Indica II	Korea	SRR1239977
Iksan438	W175	Intermediate	Korea	SRR1239978
Milyang23	W176	Indica II	Korea	SRR1239979
MR185	W177	Indica II	Malaysia	SRR1239980
Manawthukha	W178	Indica III	Philippines	SRR1239981
Shwe_Thwe_Yin_Hyv	W179	Indica II	Myanmar	SRR1239982
Bg300	W180	Indica Intermediate	Sri Lanka	SRR1239983
Bg94-1	W181	Indica Intermediate	Sri Lanka	SRR1239984
CR203	W182	Indica II	Viet Nam	SRR1239985
OM997	W183	Indica II	VietNam	SRR1239986
PSB_RC_28	W184	Indica II	Philippines	SRR1239987
PSB_RC_66	W185	Indica II	Philippines	SRR1239988
TEQING	W186	Indica Intermediate	Philippines	SRR1239989
IR68552-55-3-2	W187	Indica Intermediate	Philippines	SRR1239990
IR66897B	W188	Indica II	Unknown	SRR1239991
IR58025B	W189	Indica II	Unknown	SRR1239992
Pusa_(Basmatil)	W191	Intermediate	China	SRR1239994
ASD16	W192	Intermediate	India	SRR1239995
Dianjing	W193	Temperate Japonica	Unknown	SRR1239996
F6	W194	Indica II	Japan	SRR1239997
452	W195	Indica II	Unknown	SRR1239998
Ai-Zi-DAO	W197	Indica II	China	SRR1240000

Cultivar Name	Cultivar ID	Subpopulation	Location	Accession
Xibaizhan	C135	Indica I	Sichuan	SRR1239735
Zegu	C136	Indica I	Guizhou	SRR1239736
Cungunuo	C137	Temperate Japonica	Guizhou	SRR1239737
Lengshuigu	C138	Temperate Japonica	Yunnan	SRR1239738
WH139	C139	Indica I	Yunnan	SRR1239739
Wuzuhonggu	C140	Indica I	Yunnan	SRR1239740
Nangaogu-1	C141	Indica I	Yunnan	SRR1239741
Mowanggunei-1	C142	Indica III	Yunnan	SRR1239742
Qitougu	C143	Indica I	Yunnan	SRR1239743
Huangpinuo	C144	Temperate Japonica	Yunnan	SRR1239744
Zhenshan97B	C145	Indica I	Zhejiang	SRR1239745
Nipponbare	C146	Temperate Japonica	Japan	SRR1239746
Minghui63	C147	Indica II	Unknown	SRR1239747
9311	C148	Indica II	Hunan	SRR1239748
Zhonghua11	C149	Temperate Japonica	Beijing	SRR1239749
Balilla	C150	Temperate Japonica	Italy	SRR1239750
Nanjing11	C151	Indica I	Jiangsu	SRR1239751
02428	C152	Temperate Japonica	Jiangsu	SRR1239752
IRAT109	C153	Tropical Japonica	Unknown	SRR1239753
Heigeng2hao	C154	Temperate Japonica	Heilongjiang	SRR1239754
Erjiunan1hao	C155	Indica I	Zhejiang	SRR1239755
ChaoyangyihaoB	C156	Indica I	Hunan	SRR1239756
L301B	C157	Indica I	Hunan	SRR1239757
Guangluai15	C158	Indica I	Guangxi	SRR1239758
ZhuzhenB	C159	Indica I	Hunan	SRR1239759
Baoxie-7B	C160	Indica Intermediate	Hunan	SRR1239760
Gzhenshan97B	C161	Indica I	Sichuan	SRR1239761
Nanxiongzaoyou	C162	Indica Intermediate	Guangdong	SRR1239762
Zaoshuxianghei	C163	Indica II	Guangxi	SRR1239763
IR661-1	C164	Indica II	Unknown	SRR1239764
Nanjing11-2	C165	Indica I	Jiangsu	SRR1239765
Gui630	C166	Indica Intermediate	Unknown	SRR1239766
76--1	C167	Indica Intermediate	Unknown	SRR1239767
Xugunuo	C168	Indica Intermediate	Hunan	SRR1239768
Taizhongzailai1	C169	Indica Intermediate	Taiwan	SRR1239769
Xiangwanxian1hao	C170	Indica I	Hunan	SRR1239770
Xingguo	C171	Temperate Japonica	Jilin	SRR1239771
Zhonghua8hao	C172	Temperate Japonica	Beijing	SRR1239772
Aimakang	C173	Indica I	Sichuan	SRR1239773
JWR221	C174	Indica II	Unknown	SRR1239774
Zhengxian232	C175	Indica Intermediate	Jiangsu	SRR1239775
Chenwan3hao	C176	Indica Intermediate	Hunan	SRR1239776
Maguzi	C177	Temperate Japonica	Shanxi	SRR1239777
Aimi	C178	Indica I	Jiangxi	SRR1239778
Haobayong1	C179	Temperate Japonica	Yunnan	SRR1239779
Wanlixian	C180	Indica I	Hunan	SRR1239780
Feidongtangdao	C181	Temperate Japonica	Anhui	SRR1239781
Hengxianliangchun	C182	Indica I	Guangxi	SRR1239782
Leihuozhan	C183	Indica I	Anhui	SRR1239783
Baikezaohe	C184	Indica I	Hunan	SRR1239784
Haomake(K)	C185	Japonica Intermediate	Yunnan	SRR1239785
Sanbangqishiluo	C186	Japonica Intermediate	Yunnan	SRR1239786
Niankenuo	C187	Temperate Japonica	Guizhou	SRR1239787
Yangkenuo	C188	Temperate Japonica	Guizhou	SRR1239788
Putaohuang	C189	Temperate Japonica	Tianjin	SRR1239789
Xiangdao	C190	Indica I	Henan	SRR1239790
Younian	C191	Indica I	Guizhou	SRR1239791
Wukezhan	C192	Indica Intermediate	Fujian	SRR1239792
Zhongnong4hao	C193	Indica I	Sichuan	SRR1239793
Minbeiwanxian	C194	Indica Intermediate	Fujian	SRR1239794
Hongmisandan	C195	Temperate Japonica	Jiangxi	SRR1239795
Yuyannuo-2	C196	Temperate Japonica	Yunnan	SRR1239796
Maweinian	C197	Indica I	Guizhou	SRR1239797
Zinuo	C198	Indica Intermediate	Yunnan	SRR1239798
Beizinuo	C199	Indica I	Yunnan	SRR1239799
Biwusheng	C200	Indica I	Yunnan	SRR1239800
Yizhixiang	C201	Indica Intermediate	Fujian	SRR1239801
Xiaobaimi	C202	Indica I	Guizhou	SRR1239802
Babaomi	W198	Indica Intermediate	China	SRR1240001
Diantun502	W199	Indica Intermediate	yunnan	SRR1240002
Heiheaihui	W200	Indica Intermediate	Unknown	SRR1240003
Jiangxisimiao	W201	Indica Intermediate	Jiangxi	SRR1240004
Laohudao	W202	Indica Intermediate	Zhejiang	SRR1240005
Linjintangdao	W203	Indica Intermediate	Unknown	SRR1240006
Mengguandamagu	W204	Indica I	Unknown	SRR1240007
Pengshantieganzhan	W205	Indica I	Unknown	SRR1240008
Wumanggaonuo	W206	Indica Intermediate	Unknown	SRR1240009
Yunguang8hao	W207	Temperate Japonica	yunnan	SRR1240010
Zhongchao123	W208	Temperate Japonica	Unknown	SRR1240011
Gizal159	W209	Temperate Japonica	Unknown	SRR1240012
Khao_Daeng	W210	Indica I	Philippines	SRR1240013
Basmati370	W211	Indica II	Philippines	SRR1240014
Bhavani	W212	Indica Intermediate	India	SRR1240015
IR50	W213	Indica Intermediate	India	SRR1240016
Jhona349	W214	Aus	Pakistan	SRR1240017
Karnal_Local	W215	Aus	India	SRR1240018
Type3	W216	Indica II	India	SRR1240019
IRAT352	W217	Indica Intermediate	Indonesia	SRR1240020
TB154E-TB-2	W218	Indica Intermediate	Unknown	SRR1240021
Domsiah	W219	VI/Aromatic	Netherlands	SRR1240022
Tarommolai1	W220	VI/Aromatic	Unknown	SRR1240023
MR77_(seberang)	W221	Indica Intermediate	Philippines	SRR1240024
ir6	W222	Indica II	Pakistan	SRR1240025
At354	W223	Indica II	Sri Lanka	SRR1240026
BG304	W224	Indica II	Sri Lanka	SRR1240027
Lemont	W225	Tropical Japonica	Philippines	SRR1240028
M401	W226	Temperate Japonica	Philippines	SRR1240029
OM1706	W227	Indica Intermediate	Viet Nam	SRR1240030
OM1723	W228	Indica II	Viet Nam	SRR1240031
X21	W229	Indica II	Viet Nam	SRR1240032
X22	W230	Indica II	Viet Nam	SRR1240033
X23	W231	Indica II	Viet Nam	SRR1240034
C70	W233	Indica Intermediate	Viet Nam	SRR1240036
Q5	W234	Indica Intermediate	Viet Nam	SRR1240037
Suiyangnian	W235	Indica I	Unknown	SRR1240038
Pokhreli	W236	Indica II	Philippines	SRR1240039
Govnd	W237	Indica II	Unknown	SRR1240040
UPR191-66	W238	Indica II	India	SRR1240041
ASD18	W239	Indica Intermediate	India	SRR1240042
TGMS29	W240	Indica Intermediate	India	SRR1240043
Phalguna	W241	Indica II	India	SRR1240044
Ajaya	W242	Indica Intermediate	India	SRR1240045
Dodda	W243	Indica II	Unknown	SRR1240046
Palung_2	W244	Indica II	Philippines	SRR1240047
TKM6	W245	Indica Intermediate	India	SRR1240048
RUSTYLATE/Zhong_413	W246	Indica Intermediate	Unknown	SRR1240049
Yenfangghu	W247	Indica I	Unknown	SRR1240050
Heimichut	W248	Indica I	Unknown	SRR1240051
Teksichut	W249	Temperate Japonica	Philippines	SRR1240052
Eiko	W250	Temperate Japonica	Japan	SRR1240053
RUBIO	W251	Tropical Japonica	Philippines	SRR1240054
SADRI_RICE_1	W252	VI/Aromatic	Iran	SRR1240055
Sereendan_Kuning	W253	Tropical Japonica	Malaysia	SRR1240056
SAI-BUI-BAO	W254	Tropical Japonica	Viet Nam	SRR1240057
ZIRI	W255	VI/Aromatic	India	SRR1240058
Dacca6	W256	VI/Aromatic	United States	SRR1240059
Latisai1	W257	Tropical Japonica	Unknown	SRR1240060
Amareles	W258	VI/Aromatic	Portugal	SRR1240061
Up15	W259	Indica II	Japan	SRR1240062
Uz-Rosz_275	W260	Tropical Japonica	Philippines	SRR1240063
Ginga	W261	Intermediate	Japan	SRR1240064
Bintapan	W262	Indica I	Philippines	SRR1240065
SADAJIRA-19-303	W263	Indica Intermediate	Unknown	SRR1240066
CHOROFA	W264	Indica Intermediate	Philippines	SRR1240067
La110	W265	Indica Intermediate	United States	SRR1240068
Yunjiang35	W266	Indica III	Unknown	SRR1240069

Cultivar Name	Cultivar ID	Subpopulation	Location	Accession
Zaoxian240	C203	Indica I	Anhui	SRR1239803
Mayang_Khang	W001	Indica I	Indonesia	SRR1239804
E_B_Gopher	W002	Tropical Japonica	United States	SRR1239805
C_5560	W003	Tropical Japonica	Thailand	SRR1239806
Quinimpol	W004	Tropical Japonica	Philippines	SRR1239807
TAICHU_MOCHI_59	W005	Tropical Japonica	Taiwan	SRR1239808
WC_2811	W006	Tropical Japonica	Micronesia	SRR1239809
Criollo_Chivacoa_2	W007	Tropical Japonica	Venezuela	SRR1239810
Secano_do_Brazil	W008	Tropical Japonica	El Salvador	SRR1239811
BERLIN	W009	Indica III	Costa Rica	SRR1239812
Sel._No._388	W010	Japonica Intermediate	Uruguay	SRR1239813
SHIMIZU_MOCHI	W011	Temperate Japonica	Japan	SRR1239814
KRASNODARSKIJ_424	W012	Temperate Japonica	Russian	SRR1239815
Pergonil_15	W013	Temperate Japonica	Portugal	SRR1239816
Red_Khosha_Cerma	W014	VI/Aromatic	Afghanistan	SRR1239817
Safut_Khosha	W015	Aus	Afghanistan	SRR1239818
NORIN_11	W016	Temperate Japonica	Japan	SRR1239819
R_75	W017	Tropical Japonica	Senegal	SRR1239820
UZ_ROSZ_M38	W018	Temperate Japonica	Uzbekistan	SRR1239821
H57-3-1	W019	Japonica Intermediate	Argentina	SRR1239822
IARI_6626	W020	Aus	India	SRR1239823
Khao_Luang	W021	Japonica Intermediate	Laos	SRR1239824
C_8429	W022	Tropical Japonica	Papua New	SRR1239825
Warrangal_Culture_1252	W023	Indica III	India	SRR1239826
Padi_Pohon_Batu	W024	Tropical Japonica	Malaysia	SRR1239827
NC_1/536	W025	Aus	Pakistan	SRR1239828
Won_Son_Zo_No._11	W026	Indica I	Korea	SRR1239829
Chacareiro_Uruguay	W027	Temperate Japonica	Uruguay	SRR1239830
Doble_Carolina	W028	Aus	Uruguay	SRR1239831
Ai_Chueh_Ta_Pai_Ku	W029	Indica Intermediate	Taiwan	SRR1239832
Thang_10	W030	Indica Intermediate	Vietnam	SRR1239833
Sipirasikkam	W031	Indica I	Indonesia	SRR1239834
TJ	W032	Indica I	Guyana	SRR1239835
PD_46	W033	Indica I	Sri Lanka	SRR1239836
PATNAI_6	W034	Aus	Myanmar	SRR1239837
K8C-263-3	W035	Indica Intermediate	Suriname	SRR1239838
Chun_118-33	W036	Indica I	China	SRR1239839
Manga_Kely_694	W037	Indica Intermediate	Madagascar	SRR1239840
BLUE_STICK	W038	Temperate Japonica	Fiji	SRR1239841
Nam_Dawk_Mai	W039	Indica I	Thailand	SRR1239842
INIAP_7	W040	Indica II	Ecuador	SRR1239843
Onu_B	W041	Tropical Japonica	Zaire	SRR1239844
Red	W042	Indica Intermediate	Pakistan	SRR1239845
Dichroa_Alef_Uslkij	W043	Indica Intermediate	Kazakhstan	SRR1239846
BKN_6987-68-14	W044	Indica II	Thailand	SRR1239847
KUBANETS_508	W045	Temperate Japonica	Russian	SRR1239848
IR_9660-48-1-1-2	W046	Indica II	Philippines	SRR1239849
Jumli_dhan	W047	Japonica Intermediate	Nepal	SRR1239850
N-2703	W048	Aus	Nepal	SRR1239851
PHUDUGEY	W049	Aus	Bhutan	SRR1239852
Ak_Tokhum	W050	VI/Aromatic	Azerbaijan	SRR1239853
RP2151-173-1-8	W051	Indica II	India	SRR1239854
HB-6-2	W052	Temperate Japonica	Hungary	SRR1239855
GPNO_1106	W053	Tropical Japonica	Guatemala	SRR1239856
Toga	W054	Indica Intermediate	India	SRR1239857
Ragasu	W055	Intermediate	Taiwan	SRR1239858
Tamanishiki	W056	Temperate Japonica	Japan	SRR1239859
Grassy	W057	Tropical Japonica	Haiti	SRR1239860
Kao_Chio_Lin_Chou	W058	Indica I	Taiwan	SRR1239861
Niwahutaw_Mochi	W059	Temperate Japonica	Japan	SRR1239862
Somewake	W060	Temperate Japonica	Japan	SRR1239863
Ardito	W061	Japonica Intermediate	Italy	SRR1239864
NANTON_NO._131	W062	Tropical Japonica	Taiwan	SRR1239865

Cultivar Name	Cultivar ID	Subpopulation	Location	Accession
Yunhui72	W267	Indica Intermediate	China	SRR1240070
Wudadaozhong	W268	Indica Intermediate	South Korea	SRR1240071
IR65600-27-1-2-2	W269	Indica Intermediate	Philippines	SRR1240072
Yetuozai	W270	Indica Intermediate	Unknown	SRR1240073
P59279	W271	Indica Intermediate	Unknown	SRR1240074
Tsao_wan_ching	W272	Indica Intermediate	China	SRR1240075
Anambae_ndanggalasi	W273	Tropical Japonica	Indonesia	SRR1240076
Er_chiu_ching	W274	Indica I	China	SRR1240077
Heen_goda_wee	W275	Tropical Japonica	Sri Lanka	SRR1240078
Pathma_wee	W276	Indica III	Sri Lanka	SRR1240079
Lakkhi_kajal	W277	Aus	Bangladesh	SRR1240080
Moisdol	W278	Aus	Bangladesh	SRR1240081
Jabor_sail	W279	Aus	Bangladesh	SRR1240082
Sada_solay	W280	Aus	Pakistan	SRR1240083
Sholay	W281	Temperate Japonica	Pakistan	SRR1240084
Ma_ba_you_zhan	W282	Indica Intermediate	China	SRR1240085
Daegujo	W283	Temperate Japonica	Republic of	SRR1240086
Paikasa	W284	Tropical Japonica	Malaysia	SRR1240087
Uwi	W285	Tropical Japonica	Indonesia	SRR1240088
Garia	W286	Aus	Bangladesh	SRR1240089
AUS_449	W287	Aus	Bangladesh	SRR1240090
Dourado_Precoce	W288	Tropical Japonica	Brazil	SRR1240091
IR_661-1-140-3-117	W289	Indica II	Philippines	SRR1240092
Aichi_Asahi	W290	Temperate Japonica	Japan	SRR1240093
IR_2071-625-1-252	W291	Indica II	Philippines	SRR1240094
Khoia_boro	W292	Aus	Bangladesh	SRR1240095
IR_8	W293	Indica II	Philippines	SRR1240096
LABELLE	W294	Intermediate	United States	SRR1240097
LEBONNET	W295	Indica II	United States	SRR1240098
AUS_371	W296	Aus	Bangladesh	SRR1240099
AUS_373	W297	Aus	Bangladesh	SRR1240100
IR_36	W298	Indica II	Philippines	SRR1240101
JAYA	W299	Indica Intermediate	India	SRR1240102
NEW_BONNET	W300	Tropical Japonica	United States	SRR1240103
IR_64	W301	Indica II	Philippines	SRR1240104
KUNTLAN	W302	Tropical Japonica	Indonesia	SRR1240105
British_Honduras_Creole	W303	Tropical Japonica	Belize	SRR1240106
CPSLO_17	W304	Intermediate	United States	SRR1240107
AKITAKOMACHI	W305	Temperate Japonica	Japan	SRR1240108
BASMATI_385	W306	VI/Aromatic	Pakistan	SRR1240109
AGNO_(PSBRC28)	W307	Indica II	Philippines	SRR1240110
PSBRC82	W308	Indica II	Philippines	SRR1240111
NSICRC122	W309	Indica II	Philippines	SRR1240112
BRRI_DHAN_29	W310	Indica Intermediate	Bangladesh	SRR1240113
PR_116	W311	Indica II	Punjab	SRR1240114
CIGEULIS	W312	Indica II	Indonesia	SRR1240115
OM_2517	W313	Indica II	Viet Nam	SRR1240116
BRRI_DHAN_28	W314	Indica II	Bangladesh	SRR1240117
CIBOGO	W315	Indica II	Indonesia	SRR1240118
NEDA	W316	Indica II	Islamic Republic	SRR1240119
CIHERANG	W317	Indica II	Indonesia	SRR1240120
Qiuqianbai	W318	Indica I	Anhui	SRR1240121
Guangkexiangnuo	W319	Temperate Japonica	Guangxi	SRR1240122
Wenxiangnuo	W320	Indica Intermediate	Yunnan	SRR1240123
Laozaogu	W321	Indica III	Yunnan	SRR1240124
Lengshuinuo	W322	Indica Intermediate	Yunnan	SRR1240125
Feienuo2	W323	Japonica Intermediate	Guizhou	SRR1240126
Zimangfeie	W324	Japonica Intermediate	Guizhou	SRR1240127
Guantuibaihe	W325	Temperate Japonica	Guizhou	SRR1240128
Haolvguangnian	W326	Japonica Intermediate	Guizhou	SRR1240129
JinghuB	W327	Temperate Japonica	Anhui	SRR1240130
Sibeitichao6	W328	Intermediate	Beijing	SRR1240131
GPNO_5055	W329	Tropical Japonica	United States	SRR1240132
ASWINA_330	W330	Aus	Bangladesh	SRR1240133

This information from Zhao H et.al., 2021.

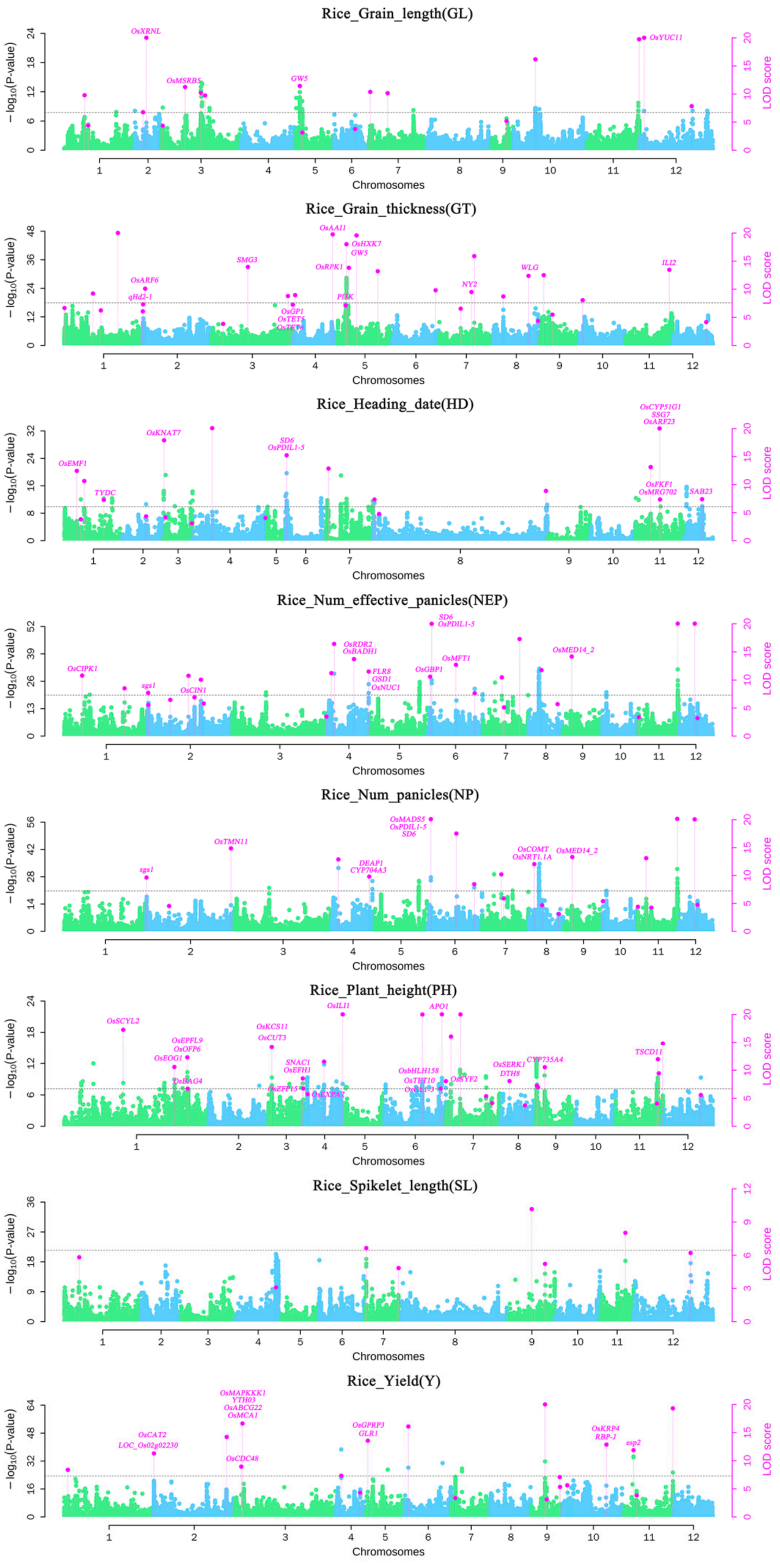

Figure S1 The Manhattan map of 8 rice traits by using the Fast3VmrMLM method. The left vertical axis shows log10(P-values) of QTNs identified through whole-genome single-marker scanning, while the right axis displays LOD values by likelihood ratio testing. The highlighted text indicates corresponding known genes.

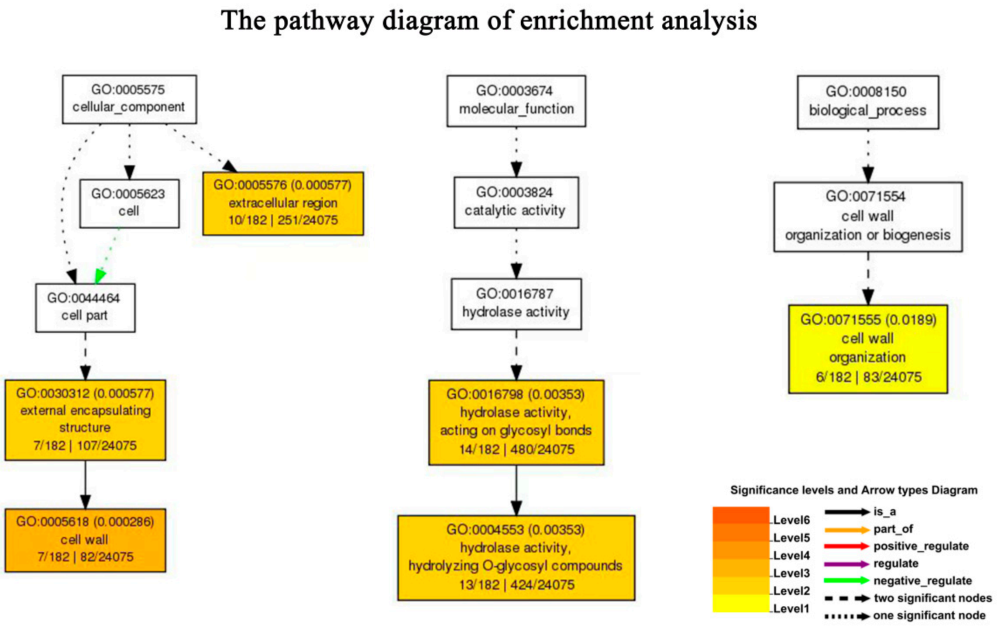

Figure S2 The graph of GO terms in biological process by enrichment analysis.
